# Analysis of Transition from Compact to Mossy Structures During Galvanostatic Zinc Electrodeposition and Its Implications for CO_2_ Electroreduction

**DOI:** 10.3390/nano15131025

**Published:** 2025-07-02

**Authors:** Pietro Altimari, Silvia Iacobelli, Pier Giorgio Schiavi, Gianluca Zanellato, Francesco Amato, Andrea Giacomo Marrani, Olga Russina, Alessia Sanna, Francesca Pagnanelli

**Affiliations:** 1 Department of Chemistry, Sapienza University of Rome, Piazzale Aldo Moro 5, 00185 Rome, Italy; s.iacobelli@uniroma1.it (S.I.); giangy.zazza@gmail.com (G.Z.); francesco.amato@uniroma1.it (F.A.); andrea.marrani@uniroma1.it (A.G.M.); olga.russina@uniroma1.it (O.R.); francesca.pagnanelli@uniroma1.it (F.P.); 2 Department of Basic and Applied Sciences for Engineering, Sapienza University of Rome Via Antonio Scarpa 16, 00161 Rome, Italy; alessia.sanna@uniroma1.it

**Keywords:** zinc electrodeposition, composite zinc electrodes, mossy morphology, CO_2_ electroreduction

## Abstract

The galvanostatic electrodeposition of zinc on carbon paper from mildly acidic solutions (ZnCl_2_: 0.05–0.1 M; H_3_BO_3_: 0.05 M) was investigated. The deposits’ growth mechanisms were analyzed through the study of the electrodeposition potential transients and the physical characterization of the electrodes synthesized by varying the current density, transferred charge, and zinc precursor concentration. The analysis reveals that the transition from crystalline to amorphous mossy deposits takes place via the electrodeposition of metallic zinc followed by the formation of oxidized zinc structures. The time required for this transition can be controlled by varying the zinc precursor concentration and electrodeposition current density, allowing for the synthesis of composite zinc/oxidized zinc electrodes with varying ratios of the oxidized to underlying metallic phases. The impact of this ratio on the electrode activity for CO_2_ electroreduction is analyzed, highlighting that composite zinc/oxidized zinc electrodes can achieve a faradaic efficiency to CO equal to 82% at −1.8 V vs. Ag/AgCl. The mechanisms behind the variations in the catalytic activity with varying morphologies and structures are discussed, providing guidelines for the synthesis of composite zinc/oxidized zinc electrodes for CO_2_ electroreduction.

## 1. Introduction

Zinc-based materials have traditionally been employed in a wide range of applications, including in the synthesis of corrosion-resistant coatings [1,2], semiconductors [3,4,5], battery anodes [6,7] and nanoparticles with antimicrobial activities [8]. In all of these applications, the achieved performance is dependent on the material morphology and structure. This has raised interest in the development of strategies for the morphology-controlled synthesis of zinc materials [9,10,11,12,13]. Solution-based methods, including chemical precipitation [9] and hydrothermal [14] routes, are preferred for the production of zinc powders, while electrodeposition is the most widely used technique for the synthesis of zinc coatings [2,15].

Compared to solution-based methods, electrodeposition offers the advantage of the immobilization of the synthesized material onto the substrate in a unique step. Further, the morphology and structure of zinc deposits can be easily controlled by varying the working electrode potential and/or the composition of the electrolyte bath. As these parameters are varied, zinc deposits with widely different morphologies are formed, ranging from compact crystalline plates [16,17] to irregular, branched-like mossy [18,19,20] and dendritic [21] structures. This rich polymorphism has contributed to an increase in the scientific interest in zinc electrodeposition [13,22,23]. In this framework, numerous theoretical and experimental studies have been reported aimed at unveiling the mechanisms of zinc electrodeposition, ranging from the analysis of the electrochemical nucleation [24,25,26] to the study of the morphology evolution [27,28,29].

This research on the fundamental aspects of electrodeposition is crucial to tailoring the morphologies and structures of zinc deposits. In this regard, it is important to note that markedly different zinc morphologies can be targeted depending on the selected application. For example, the research on zinc rechargeable batteries is focused on the identification of electrode designs and electrolytes preventing the formation of dendritic and mossy structures, which can penetrate the separator and induce the non-uniform dissolution of the anode during the battery discharge [6,7,30]. However, this perspective is reversed if the application of zinc electrodes in electrocatalytic processes is analyzed. In this case, the maximization of the specific surface and the introduction of surface defects are targeted, which have increased the interest in branched-like morphologies [31,32].

This reversal of the electrode design perspective is supported by the numerous studies that have addressed the application of zinc electrodes to the catalysis of CO_2_ electroreduction (ERC) [33,34,35]. In this application, silver is the state-of-the-art catalyst for the production of CO, with achieved CO selectivity values typically larger than 90% [36]. However, the elevated costs of this material have motivated interest in research on alternative metal-based catalysts. Here, zinc is a competitive alternative. Zinc is a widely available and affordable metal, and it can easily achieve selectivities to CO only slightly lower than those of silver [36], provided that the morphology and structure are effectively controlled [37,38,39].

For this purpose, different electrodeposition strategies have been developed that rely on the optimization of the potential/current temporal evolution. Zinc dendrites were synthesized by galvanostatic electrodeposition [40], while a porous network of zinc nanoparticles was obtained by pulsed electrodeposition [41]. To increase the specific surfaces of deposits, bubbling electrodeposition was implemented, yielding metallic foams with hierarchical porosity [37,42,43]. Alternatively, the modification of the electrolyte bath has been exploited. In this framework, by varying the concentration of cetrimonium bromide added to the electrodeposition bath, (Qin et al., 2018) [44] could purposefully modify the texture of the deposits, contributing to improvements in the catalytic activity, while the dissolution of different gases in the electrodeposition bath was exploited by (Guo et al. 2021) [45] to control the morphologies of synthesized zinc nanostructures. More recently, the co-electrodeposition of zinc and different metals was investigated [46,47,48]. In this framework, Zn/Cu electrodes with different morphologies were obtained in the form of alloys or heterostructures [49,50,51,52].

In addition to the use of metallic zinc, several studies have proposed employing zinc electrodes in oxidized form [36,53,54,55,56]. These can undergo reconstruction under the application of cathodic potentials through partial dissolution and redeposition, yielding increased specific surfaces and higher numbers of defects, which can enhance the catalytic activity [36]. Reconstruction mediated by the electrochemical reduction of oxidized zinc can be achieved separately [57], or it can take place within the electrocatalytic reactor, during the ERC test [58]. In the latter case, oxidized zinc species were found to persist at the cathodic potential values sustaining the ERC, improving the selectivity towards carbon monoxide [58].

As far as electrochemical methods are concerned, the reduction of nitrates in the presence of a zinc precursor has been exploited [24,57,59]. In the application of this method, the generation of OH^−^ is sustained by nitrate reduction at potentials lower than the zinc redox potential, which increases the local pH and induces the precipitation of zinc hydroxide nanoflakes, yielding a mossy morphology. However, a key limitation of this method is that it exclusively yields oxidized zinc (hydroxide and/or oxide), which can increase the charge transfer resistance.

An alternative electrochemical route to the synthesis of metal oxidized nanostructures is the metal electrodeposition at potential values sustaining the co-reduction of water [60,61,62,63,64]. Water reduction can increase the pH near the electrode, shifting the system to conditions wherein the metal hydroxide becomes thermodynamically stable. If this is the case, the precipitation of metal hydroxide can take place at the electrode surface. The occurrence of precipitation is determined by the interplay between the kinetics of the metal ion reduction and hydrogen evolution reaction (HER). Compared to the nitrate reduction method, the co-reduction of water allows for simultaneously sustaining the electrochemical reduction of the metal ion and the precipitation of metal hydroxide. Composite metal/metal hydroxide structures can thus be synthesized, which yield reduced charge transfer resistances [61]. The method has been implemented to fabricate cobalt [60,62,64] and nickel [61,65] electrodes for supercapacitors and electrocatalytic processes, while no study has systematically investigated its application for the synthesis of composite zinc/zinc oxide electrodes. In addition, no previous study has evaluated how the transition from compact metallic zinc structures to zinc nanoflakes during electrodeposition can affect the catalytic activity for the ERC.

Yet, previous studies relying on different synthesis routes have highlighted that composite zinc/oxidized zinc electrodes can outcompete compact metallic and non-porous zinc oxide electrodes in the ERC [36,66,67]. While the metallic core can enforce reduced charge transfer, the high area of flakes with nanoporous structures increases the access to the electron-deficient active sites, which have been found to enhance the adsorption of ERC intermediates (*COOH) [36]. In addition, zinc dissolution and redeposition mediated by the partial reduction of oxidized zinc species during the ERC can increase the number of highly uncoordinated sites and oxygen vacancies, which can further enhance the catalytic activity [36,54].

Motivated by these experimental findings, the present study aimed to investigate the transition from compact to mossy structures during the galvanostatic electrodeposition of zinc on carbon paper, and to evaluate the catalytic activity towards the ERC achieved with the different morphologies identified. An experimental analysis is reported to evaluate the influence of the electrodeposition parameters on the deposit morphology and structure. In this way, the conditions enabling the synthesis of electrodes with the following morphologies were identified: compact metallic zinc particles, oxidized mossy structures, and composite deposits resulting from the growth of oxidized mossy structures on metallic zinc. These electrodes were thoroughly characterized (XPS, Raman, XRD, XRD tomography) and tested as cathodes to run the ERC, allowing for the derivation of indications regarding the impact of the morphology and structure on the catalytic activity.

## 2. Materials and Methods

### 2.1. Electrochemical Synthesis and Characterization

The catalytic electrodes were synthetized by zinc electrodeposition on a carbon paper foil (Sigracet GDL 39 BC, SGL Carbon, Wiesbaden, Germany) with a geometric area of 4 cm^2^. The electrodeposition bath consisted of a zinc chloride solution (ZnCl_2_) (VAvantor (WR chemicals, Leuven, Belgium), with 0.5 M of boric acid (H_3_BO_3_) (Sigma-Aldrich, Steinheim, Germany), which was used to buffer the pH. This can prevent the pH from rapidly increasing and inducing zinc hydroxide precipitation without the previous formation of metallic zinc. The electrodeposition was carried out under galvanostatic conditions in a three-electrode cell configuration by employing the carbon paper as the working electrode, a counter electrode of graphite (T99%, hermo Scientific, Karlsruhe, Germany) and an Ag/AgCl reference electrode (3 M KCl) (Fluka Analytical, Seelze, Germany). The electrolyte solution was maintained at 25 °C under magnetic stirring. Electrodeposition tests were performed via an Ivium-Stat potentiostat (Ivium Technologies B.V., Eindhoven, The Netherlands).

To evaluate the impact of the electrodeposition parameters on the deposit morphology, four different electrodes were synthesized by varying the concentration of the zinc precursor in the electrolyte bath (0.1, 0.05 M ZnCl_2_) and the electrodeposition current density (2, 4 mA/cm^2^) while maintaining a transferred charge density equal to 2 C/cm^2^. In this way, the electrodeposition parameters yielding compact and mossy morphologies were identified. Successively, for two sets of the electrodeposition parameters yielding, respectively, the formation of compact and mossy structures at 2 C/cm^2^, electrodeposition tests with the transferred charge increased to 20 C/cm^2^ were carried out. To explain the impact of the current density and zinc precursor concentration on the morphologies and structures of the zinc deposits, the potential transients recorded during the galvanostatic electrodeposition experiments were recorded and analyzed.

Cyclic voltammetry was carried out from ZnCl_2_, 0.1 M, and H_3_BO_3_, 0.05 M, with a scanning rate equal to 10 mV/s under the same conditions as those used for the synthesis but without magnetic stirring.

### 2.2. Physical and Chemical Characterization

The morphology, size and spatial distribution of the electrodeposited zinc structures were characterized by sfield emission canning electron microscopy (FESEM) (AURIGA, model Zeiss), Oberkochen, Germany.

To obtain a three-dimensional reconstruction of the samples, the tomograph Xradia Versa 610 of ZEISS was used. It consisted of an X-ray source, a sample holder, rotating over 360°, and the detector. The detector consisted of a charge-coupled device (CCD), onto which the X-ray from the source converged after passing through the sample and the signal was recorded. The instrument was equipped with objectives that allowed for the optical and geometric magnification of the sample. The image was obtained by the absorption of the X-rays from the sample. The operating principle of the instrument was based on the Lambert–Beer law. The images collected were two-dimensional and three-dimensional. The X-rays from the tomograph were absorbed by the electrodes, and the instrument processed the voxels, which, similar to the pixels for two-dimensional images, divided the three-dimensional images into volumes. The computerized projection of the sample was returned from the processing. The measurement was performed on a small central portion of the electrode to obtain a high-resolution tomographic image. The instrument processed the analysis, calculating the surface value of the zinc. By knowing the density of the zinc (7.13 g/cm^3^), it was possible to quantify the volume of the metal from the surface.

The electrodes’ crystallinity was investigated by XRD analysis. For this purpose, a Bruker AXS D8 ADVANCE (Bruker AXS, Karlsruhe, Germany)with Mo Kα radiation (λ = 0.71Å) was employed. XRD patterns of the different electrodes were analyzed and compared with the carbon paper support.

Raman spectra were run at r.t. in backscattering geometry with an inVia Renishaw micro-Raman spectrometer equipped with an air-cooled CCD detector and super-Notch filters. An Ar+ ion laser (λlaser = 514 nm) was used, coupled to a Leica DLML microscope with a 20× objective. The resolution was 2 cm^−1^, and the spectra were calibrated using the 520.5 cm^−1^ line of a silicon wafer. Each spectrum was collected at 50% of laser power, 10 s of spectral acquisitions and 20 scans.

To study the superficial composition of the electrodes, X-ray photoelectron spectroscopy (XPS) (Omicron NanoTechnology Multiprobe system) was used. The samples’ ionization was excited using a monochromatic Al Kα (hν = 1486.7 eV) X-ray source. The wide (low-resolution) spectra were recorded at a pass energy of 50 eV and a take-off angle of 21°. For high-resolution narrow-scan analyses, a pass energy of 20 eV was used. The experimental spectra were theoretically reconstructed fitting the secondary electrons’ background to a Shirley function and the elastic peaks to pseudo-Voigt functions. XPS atomic ratios between relevant core lines were estimated from experimentally determined area ratios (±10% associated error) corrected for the corresponding Scofield cross sections [68] and for a square root of the photoelectrons’ kinetic energies.

The amount of metal deposited on the electrodes was quantified by atomic absorption spectrophotometry (AAS) (ContraAA 300–Analytik Jena AG, Jena, Germany) after the acid digestion of the electrodes in aqua regia using nitric acid (HNO_3_, 65%) (AnalaR NORMAPUR^®^, VAvantor, Fontenay-sous-Bois, France) and hydrochloric acid (HCl, 37%) (EMSURE^®^, Merk, Darmstadt, Germany). The efficiency of the zinc electrodeposition was then quantified as the ratio of the electric charge associated with the mass of deposited zinc, calculated by using Faraday’s law, to the total charge transferred during the electrodeposition.

### 2.3. Electrocatalytic Tests

Chronoamperometric tests were carried out to study the selectivity of the electrodes towards gaseous products (H_2_ and CO) and the stability of the electrodes over time. For this purpose, an H-like cell was used, composed of two compartments separated by a proton exchange membrane, Nafion (N-117 membrane, 0.180 mm thick, >0.90 meq/g exchange, VWR). A three-electrode configuration was adopted, with the zinc electrodes and a graphite electrode used as the cathodes and anodes, respectively, and an Ag/AgCl saturated electrode used as the reference electrode. The electrolyte used was a 0.5 M potassium bicarbonate solution (KHCO_3_, ≥99.5%) (AnalaR NORMAPUR^®^, VWR). At the beginning of the tests, the electrolyte solution was saturated by bubbling CO_2_ for 30 min at a potential of −1.3 V (vs. Ag/AgCl). The electroreduction of CO_2_ was then carried out under magnetic stirring at −1.8 V and −2.0 V (vs. Ag/AgCl). 

The Clarus 690 gas chromatograph with a thermal conductivity detector (TCD) (PerkinElmer, Shelton, WA, USA) was used for the analysis of the gaseous products (H_2_ and CO). The instrument had a Restek operating column of 2 m, with a carbon molecular sieve. The gas carrier used was Argon (Ar, 99.99%) (SOL Spa, Monza, Italy). The sampling was performed continuously by loop, with a fixed-volume valve of 500 μL. The faradaic efficiencies (FEs) of the H_2_ and CO were computed as follows:

(1) $${FE}_{i}=\frac{G\cdot c_{i}\cdot z_{i}\cdot F}{I}$$

where c_i_ (i = H_2_, CO) is the molar concentration of the gaseous product (i) derived from the relative chromatogram peak’s area, z_i_ is the number of electrons exchanged per mol of product (i), F = 96485.3 C/mol is the Faraday constant, and G and I are the gas flow rate pumped through the cell and the current density, respectively. The flow of CO_2_ was maintained constant at 10 mL/min. For the electrocatalytic tests, each fabricated electrode was used only at one potential. In addition, at least two different electrodes of the same type were prepared and separately used for each potential.

The potential values reported throughout this article are quantified with respect to the Ag/AgCl reference electrode.

## 3. Results

### 3.1. Synthesis of the Zinc Electrodes by Electrodeposition

**Figure 1:**
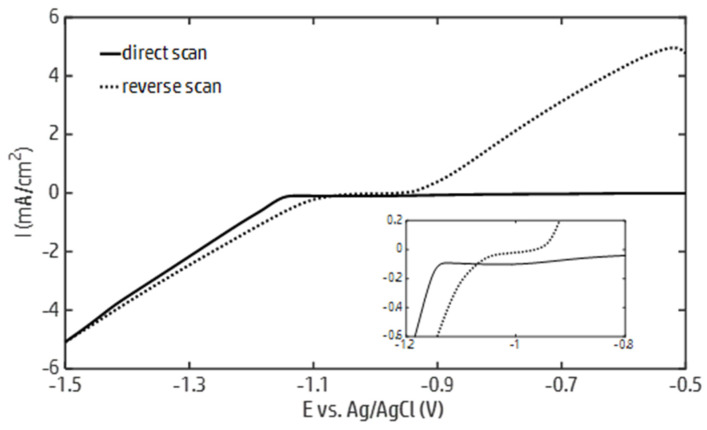
Current–potential curves generated by cyclic voltammetry from a zinc chloride solution (ZnCl_2_ 0.1 M, H_3_BO_3_ 0.05 M) with a scanning rate equal to 10 mV/s.

In order to identify the conditions that allow for the electrodeposition of metallic zinc followed by the precipitation of zinc hydroxide, cyclic voltammetry in a 0.1 M ZnCl_2_, 0.05 M H_3_BO_3_ solution was carried out. The recorded current–potential curves are reported in Figure 1. As the potential decreased during the forward scan from −0.5 V, a sharp increase in the cathodic current was observed at −1.15 V, marking the onset of the zinc electrodeposition. As the potential decreased up to −1.5 V, the current steadily increased. In this potential interval, the overlapping of the water and zinc electrochemical reduction was observed. During the reverse scan, a crossover was observed, after which the zinc electrodeposition was sustained up to a value close to the zinc redox potential (~−0.98 V). The crossover can be attributed to the formation of zinc deposits during the direct scan, which decreased the overpotential of the zinc electrodeposition.

In accordance with the Pourbaix diagram of zinc [69], the potential below which the formation of oxidized zinc species can take place in a 0.1 M ZnCl_2_ solution decreases from approximately −0.98 to −1.4 V as the pH varies from 5 to 14. Based on the data reported in Figure 1, these potential values can be maintained by running the galvanostatic electrodeposition of zinc up to a current density of about 4 mA/cm^2^. Within this current density range, the formation of zinc hydroxide is allowed, provided that the HER increases the local pH at the electrode surface to the boundary of the zinc predominance region. Therefore, galvanostatic electrodeposition tests were carried out at current densities of 2 and 4 mA/cm^2^. The effect of the zinc concentration on the morphologies and structures of the deposits was investigated by running the electrodeposition tests in 0.05 and 0.1 M ZnCl_2_ solutions.

**Figure 2:**
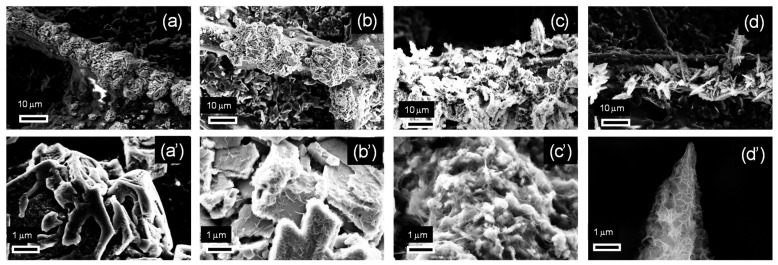
SEM images of the electrodes fabricated with varying zinc precursor concentrations and electrodeposition current densities with a transferred charge equal to 2 C/cm^2^: (**a**,**a′**) 0.05 M, 2 mA/cm^2^; (**b**,**b′**) 0.05 M, 4 mA/cm^2^; (**c**,**c′**) 0.1 M, 2 mA/cm^2^; (**d**,**d′**) 0.1 M, 4 mA/cm^2^. Figures (**a′**–**d′**) report the magnifications of the images reported in Figures (**a**–**d**), respectively.

The SEM images of the zinc deposits obtained with these electrodeposition parameters are reported in Figure 2. Variations were observed in the morphology as the electrodeposition parameters changed. The most significant effect was induced by varying the concentration of zinc in the electrolyte solution: at 0.05 M (Figure 2a,a′,b,b′), compact zinc structures with clearly defined geometries were found, while nanoflakes appeared at 0.1 M (Figure 2c,c′,d,d′), yielding a mossy morphology. Nonetheless, increasing the current density appears to partially promote the emergence of ordered structures. This can be verified by observing that, at 0.1 M, only mossy structures are found at 2 mA/cm^2^ (Figure 2c,c′), while cusped platelets can be distinguished at 4 mA/cm^2^ (Figure 2d,d′). Likewise, at 0.05 M, a transition from compact particles (Figure 2a,a′) to two-dimensional structures with a characteristic length around 1–2 μm (Figure 2b,b′) is observed with the increase in the current density from 2 to 4 mA/cm^2^. Under these conditions, even though less distinguishable compared to Figure 2c,d, mossy structures can already be identified by a close inspection of the deposit surface (Figure 2b′).

The results reported in Figure 2 apparently contradict the theory of electrocrystallization [70]. In such a process, compact metallic structures are typically formed under kinetically controlled growth, while irregular dendritic and fractal structures are formed by diffusion-limited aggregation with an increasing cathodic potential or a decreasing metal precursor concentration [71]. This is the opposite of the behavior described in Figure 2, where increasing the concentration of the zinc precursor is found to favor the transition to an irregular mossy morphology. An explanation for this apparent contradiction is that the irregular mossy structures shown in Figure 2b,d are formed through zinc hydroxide precipitation. In fact, zinc electrodeposition takes place at potentials sufficiently large to sustain the co-reduction of water, which can induce an alkalinization of the electrolyte solution at the electrode surface. Under these conditions, an increase in the zinc ion concentration can promote the precipitation of zinc hydroxide at the electrode surface. This analysis is consistent with previous studies, where it was shown that the formation of mossy structures can be achieved through the precipitation of zinc hydroxide, while compact structures are typically obtained by the electrodeposition of metallic zinc [72].

**Figure 3:**
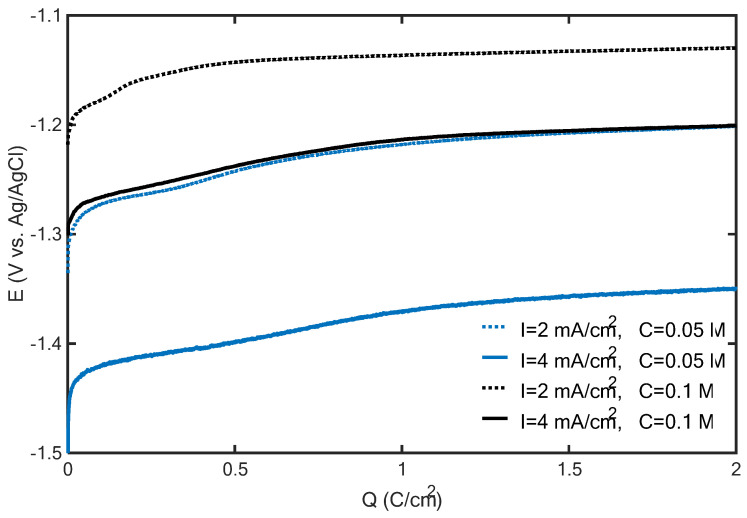
Potential transients recorded during the galvanostatic electrodeposition of zinc on carbon paper from ZnCl_2_ solutions with 0.05 M H_3_BO_3_ (blue and black lines correspond to 0.05 and 0.1 M ZnCl_2_ concentrations, respectively, while dashed and solid lines correspond to 2 and 4 mA/cm^2^, respectively).

A physical characterization of the deposits supporting this conclusion is reported in the following section. However, before illustrating this characterization, further insights into the deposit growth mechanisms can be derived by analyzing the potential transients recorded during the galvanostatic electrodeposition of the structures shown in Figure 2. These potential transients are plotted versus the amount of charge transferred during electrodeposition in Figure 3. The potential becomes less negative as the zinc concentration is increased and/or the current density is decreased. However, the zinc concentration mainly contributes to determining the potential: irrespective of the current density, the potential curves with the 0.1 M zinc concentration are both above the two potential curves recorded with 0.05 M. Since the appearance of oxidized zinc deposits is promoted, from the thermodynamic viewpoint, by decreasing the cathodic potential and increasing the zinc concentration, it can be argued that increasing the zinc concentration from 0.05 to 0.1 M can promote the formation of zinc oxidized species more effectively than decreasing the current density from 4 to 2 mA/cm^2^. This corroborates the hypothesis that the appearance of mossy structures at 0.1 M (Figure 2) can be explained by the formation of zinc oxidized species.

In accordance with the proposed analysis, the formation of flakes is determined by a shift in the electrode surface conditions (pH, potential and zinc concentration) to the region of predominance of oxidized zinc species (thermodynamic control). When decreasing the zinc concentration and/or increasing the current density, a more negative potential is required, which can favor the nucleation of metallic zinc in the early electrodeposition stage (kinetic control). However, as the surfaces of zinc deposits increase, the potential required to maintain the imposed current density becomes progressively less negative, which, again, can shift the system to the region of predominance of zinc oxidized species (thermodynamic control). In this way, composite zinc/oxidized zinc electrodes can be synthesized.

It is worth noting here that despite the observed quantitative differences, the potential transients shown in Figure 3 are qualitatively identical. In particular, irrespective of the current density and zinc concentration, as the transferred charge increased, the potential rise progressively slowed down, and a barely varying potential was eventually achieved. Under this latter condition, it was found that the HER is the main faradaic process. Via the acid digestion of the electrodes and an analysis of the zinc concentration in solution, it was found that, with a transferred charge of 1 C/cm^2^, the zinc deposition yield (i.e., the percentage of the transferred charge that can be imputed to zinc reduction) ranged between 65% and 92%, corresponding to the electrodeposition experiments with the highest and lowest potential curves in Figure 3, respectively. However, the deposition yield significantly decreased as the transferred charge increased: at 20 C/cm^2^, zinc deposition yields of 33% and 23% were found for the experiments corresponding to the highest and lowest curves in Figure 3, respectively. These results suggest that, as the duration of the electrodeposition increases, the HER covers a progressively increasing fraction of the selected current density.

As the HER partial current density increases, the zinc concentration at the electrode becomes closer to that in the bulk, while an increase in the surface pH can be predicted. These conditions increase the likelihood of zinc hydroxide precipitation. Accordingly, the observation that the HER accounted for most of the current density at the end of the experiment suggests that zinc hydroxide precipitation can be achieved not only at a 0.1 M zinc concentration (Figure 2b,c) but also at a 0.05 M zinc concentration, provided that the transferred charge (equivalently, the duration of the electrodeposition) is increased. To obtain a preliminary quantitative estimation, it can be observed that with the current density (J_0_) entirely covered by the HER, in the absence of buffering species, the following stationary value is achieved for the differential acidity (x = [H_3_O^+^] − [OH^−^]) at the electrode surface within times of the order of δ^2^/D_OH−_ [73,74]:

(2) ξ_s_ = ξ_∞_ + J_0_δ/(FD_OH−_)

where the subscripts s and ∞ denote the values at the electrode surface and in the bulk, respectively, and δ, D_OH_- and F are the thickness of the boundary diffusion layer, the OH^−^ diffusion coefficient and the Faraday constant, respectively. It can then be found that, at the electrode surface, a pH equal to 11.5 is achieved within a time of the order of 1.2 s at the lower electrodeposition current density of 2 mA/cm^2^, δ = 0.08 mm [75] and [OH^−^]_∞_ = 2 × 10^−9^ mol/L (corresponding to the measured bulk pH of 5.3) and a D_OH−_ of 5.3 × 10^−9^ m^2^/s [76]. Since the pH rise was buffered by boric acid in the present study, the electrodeposition of zinc in metallic form could be sustained over wider time intervals. However, different studies have demonstrated that boric acid has the effect of delaying the pH increase, and that hydroxide precipitation can eventually occur [77,78].

**Figure 4:**
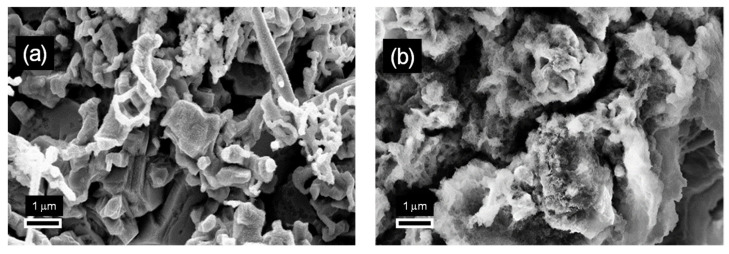
Morphology changes observed with increasing electrodeposition charge from 2 to 20 C/cm^2^; SEM images on the left (**a**) and on the right (**b**) show the change observed for the electrodes of Figure 2b,b′ and Figure 2c,c′, respectively.

To verify this prediction, SEM images of the electrodes obtained at current densities and zinc concentrations corresponding to the lower and upper potential curves shown in Figure 3, with the transferred charge increased to 20 C/cm^2^, are reported in Figure 4. While the mossy morphology of Figure 2c,c′ was maintained (Figure 4b), the compact zinc nanostructures identified in Figure 2b,b′ were no longer distinguishable when the transferred charge was increased to 20 C/cm^2^ (Figure 4a). Here, mossy structures with irregular morphologies were found to cover the previously formed zinc deposits. This suggests that mossy structures are formed by the transition from compact structures, and that the electrodeposition charge at which this transition takes place varies depending on the electrodeposition current density and the concentration of zinc in the electrolyte solution.

To validate the illustrated analysis of the electrodeposition mechanisms and evaluate how the transition from a layer to mossy morphology can affect the catalytic activity for the ERC, four different electrodes were further characterized and utilized to run electrocatalytic tests. The electrodes were selected to assess the influence of the relative weights of the compact and mossy structures in the deposits. For this purpose, the electrode corresponding to Figure 2b, for which compact layer structures were found, and the electrode corresponding to Figure 2c, for which a mossy morphology was already observed at 2 C/cm^2^, were selected. To simplify the notation, these electrodes are hereafter referred to as L2 and M2, with L and M denoting the layer and mossy morphologies, respectively, and the reported number referring to the electric charge transferred during the electrodeposition. The electrodes obtained by extending the synthesis of L2 and M2 to a transferred charge of 20 C/cm^2^ were then selected to analyze the activities of the composite layer/mossy structures and evaluate the effect of increasing the amount of mossy deposits. By following the introduced nomenclature, these two latter electrodes are referred to as L20 and M20. In what follows, a detailed physical characterization of these electrodes is reported.

### 3.2. Physical Characterization

In order to evaluate the intrinsic catalytic activity of the different morphologies, the surfaces and volumes of the zinc deposits were measured for each of the selected electrodes. For this purpose, a three-dimensional reconstruction was obtained by tomographic analysis. Three-dimensional axonometric views showing the distribution of zinc deposits along the fibers of the carbon paper are reported in Figure S1 (Supplementary Materials). Both at 2 and 20 C/cm^2^, larger structures are found for the layer electrodes compared to the mossy electrodes, confirming the indication of the SEM images. This can be explained by the fact that the early precipitation of hydroxides taking place for mossy electrodes can hamper the growth of the deposits compared to the layer electrodes [77]. The surface areas and volumes of the zinc deposits determined by processing the data from the tomographic analysis are reported in Table 1.

The M electrodes exposed a lower zinc surface compared to that of the L ones. This effect should be partly imputed to the lower amount of deposited mossy structures. However, in contrast to what was expected, the data reported in Table 1 evidence that the mossy structures were characterized by a lower surface-to-volume (S/V) ratio compared to that of the compact structures. It is hypothesized that this result could be imputed to the heterogeneous distribution of the mossy deposits forming through zinc hydroxide precipitation. Indeed, both in the SEM images and in the 3D tomographic reconstruction, along with zinc nanoflakes, larger deposits can be identified, which may contribute to reducing the S/V ratio. As the amount of deposited zinc was increased by modifying the transferred charge to 20 C/cm^2^, differences between the S/V ratio for both the L and M electrodes were reduced, which can be explained by the overlapping of the growing zinc structures.

**Figure 5:**
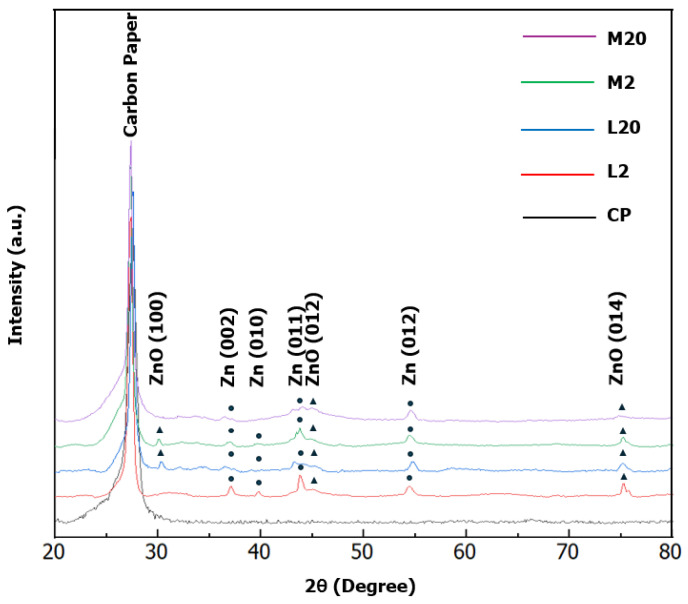
XRD spectra of the fabricated electrodes compared with that of the carbon paper (CP) support.

To derive information about the structure of the deposits, an XRD analysis was carried out. The XRD spectra of the M and L electrodes are reported in Figure 5. Notably, the analysis of the XRD data confirms that the bulk of the deposits is, for all the fabricated electrodes, mainly composed of metallic zinc, which exposes the (002) and (101) facets. This confirms that electrodeposition initially proceeds through the electrochemical reduction of zinc ions from the electrolyte solution. Here, it is worth remarking that, in accordance with previous theoretical and experimental studies [17,44], variations in the texture coefficients of the two facets (101) and (002) can affect the catalytic activity of the electrode towards the ERC. A preferential exposition of (101) facets can lower the cathodic potential required to sustain the ERC and increase the energy barrier that must be overcome to enforce the HER, thereby increasing the selectivity to CO. The (002) facet favors, in contrast, the HER. Therefore, an analysis of the XRD data was performed to evaluate the exposition of the facets (101) and (002) by the different electrodes.

In the XRD patterns of the samples L2 and M2, the intensity of the (002) reflection relative to the (101) reflection is approximately 80%. This is significantly higher than the 40% expected from the crystalline phase of h-Zn. However, the ratio is preserved for both samples, which suggests equilibrated crystal growth. Consequently, based on the only analysis of texture, the two samples were not expected to exhibit different catalytic activities in the ERC process. To extend the analysis, we evaluated the total crystallinity and crystallite size. Both the L2 and M2 samples exhibited total crystallinities of approximately 85%. However, L2 appeared to be better structured with a higher intensity of diffraction peaks and their better-defined profiles. The crystallite sizes determined for the (002) reflection were 15 nm for M2 and approximately 21 nm for L2. The crystallite sizes for the (101) reflection show only a slight variation, being 20 nm for M2 and 21 nm for L2.

In contrast, the pattern for L20 shows the reduced indication of a crystalline structure, primarily a very weak feature for the (101) reflection, as opposed to the M20 sample, which is completely amorphous. This analysis confirms that increasing the transferred charge leads to the appearance of an amorphous irregular phase on top of the metallic phase formed during the early stage of electrodeposition, which can be explained by the precipitation of zinc hydroxide.

**Figure 6:**
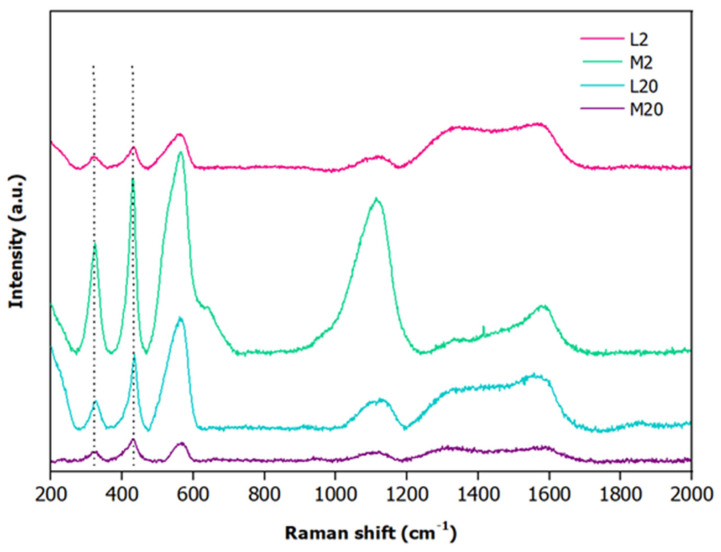
Raman spectra of L2 (pink), M2 (green), L20 (cyan) and M20 (purple) electrodes.

To gain information on the surface chemical composition of the fabricated electrodes, Raman spectroscopy was carried out (Figure 6). The Raman spectra (Figure 6) obtained for the electrodes after excitation with the 514 nm laser line clearly displayed, in all the samples, the presence of oxidized species of zinc on their surfaces owing to the presence of the peaks localized at ≈326 cm^−1^ and ≈430 cm^−1^ (dashed lines) that are associated with the second-order scattering E_2_^high^-E_2_^low^ and E_2_^high^ vibrational modes, respectively [79,80]. Furthermore, the spectra show a broad band in the spectral range between 500 and 615 cm^−1^ whose relative intensity is dependent on the crystallite size, as reported in the literature [80]. Finally, in all the spectra, the Raman signals detectable in the spectral range between 1300 and 1600 cm^−1^ are ascribable to the carbon paper.

**Figure 7:**
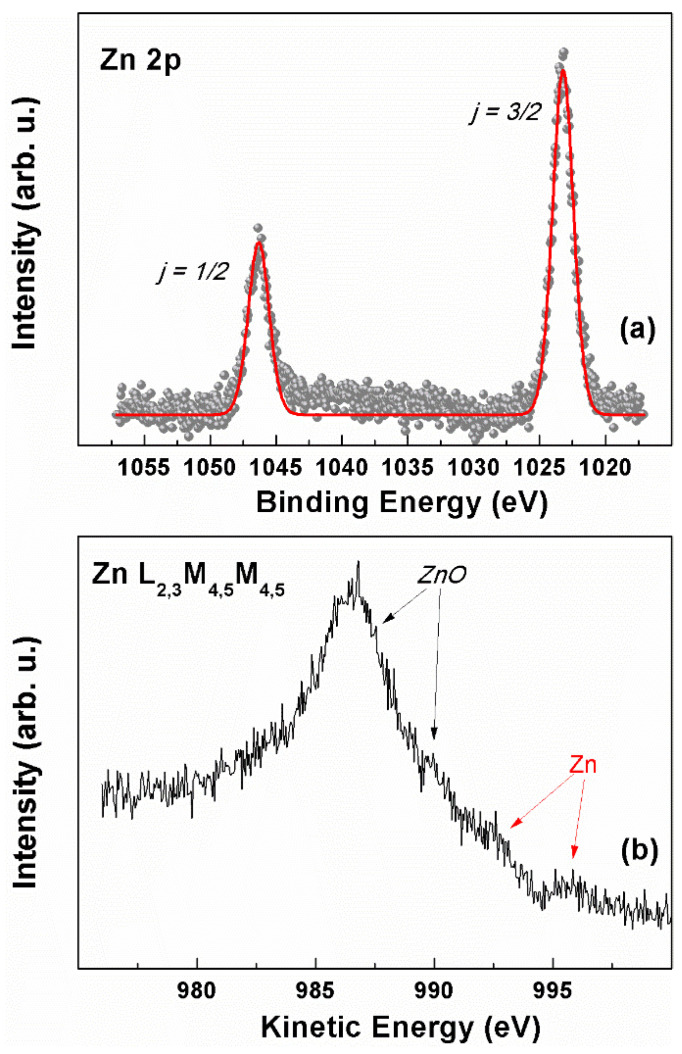
XPS characterization of the M2 sample: (**a**) Zn 2p region; (red curve is obtained by fitting the reported data (**b**) Zn L_2,3_M_4,5_M_4,5_ Auger region.

The surface composition of the electrodes was further investigated by XPS spectroscopy. The Zn 2p ionization region of a representative electrode sample, M2, is shown in Figure 7a. A spin-orbit split doublet (ΔE_SO_ = 22.9 eV) is present, with the main j = 3/2 component falling at 1023.4 eV. Since the analysis of the Zn 2p photoionization region does not allow for discerning between metallic zinc and zinc oxide due to their similar binding energy positions [81,82,83], the chemical nature of the zinc-based deposits on the electrode was investigated in the Zn L_2,3_M_4,5_M_4,5_ Auger region (Figure 7b), thereby determining the corresponding modified Auger parameter (α′) [83]. This value corresponds to the sum of the binding energy of the Zn 2p_3/2_ peak and the kinetic energy of the Zn L_3_M_4,5_M_4,5_ Auger signal, and it has the advantage of avoiding problems due to possible charge build-up upon ionization while maintaining the high chemical environment sensitivity typical of Auger electrons. Here, the obtained value was α′ = 2009.9 eV, which matches the literature data reported for ZnO [81,84]. Besides the main features attributable to ZnO (at 986.6 and ~990 eV KE), indicated with the black arrows in Figure 7b, a close inspection of the line shape of the Zn L_3_M_4,5_M_4,5_ Auger signal allows for the identification of a couple of weak bumps around 992.7 and 995.8 eV KE (indicated with the red arrows in Figure 7b), which can be associated with residual traces of freshly electrodeposited metallic Zn [82]. Argon ion sputtering could be used to further evaluate the presence of subsurface metallic zinc. However, the thorough optimization of this technique is needed to avoid the modification of the zinc oxidation state [85].

The presence of ZnO is in line with the results from the XRD and Raman spectroscopy, and the XPS suggests that electrodeposited Zn layers are quickly covered with a shell of ZnO/Zn(OH)_2_, with a thickness close to the escape depth limit of Zn 2p photoelectrons, which hampers the detection of the presence, if any, of the metallic core with this technique.

By means of the XPS peak areas, a semi-quantitative analysis was carried out to evaluate the percentage amount of Zn relative to the total carbon. This analysis confirmed that M20 and L20 were the electrodes with the largest zinc coverage, as reported in Table 2. This finding is coherent with the larger charge density applied during electrodeposition compared to the L2 and M2 samples. Notably, at both 2 and 20 C/cm^2^, the mossy electrodes were characterized by a Zn/C ratio larger than that of the electrodes with the compact layer morphology. This result suggests that the earlier formation of zinc oxidized species ensures a more extended coverage of the carbon fibers.

### 3.3. Electrocatalytic Performancess

**Figure 8:**
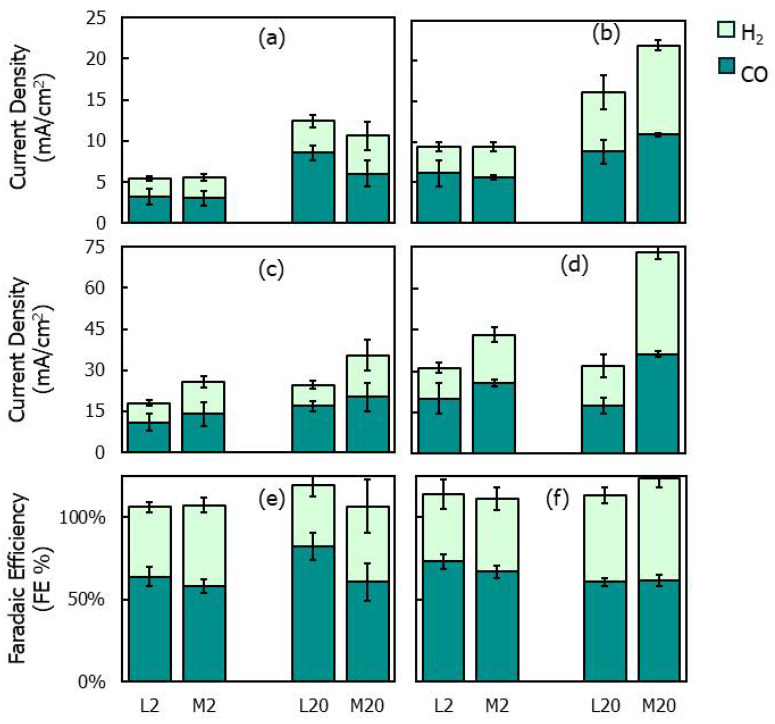
(**a**–**d**) Partial current densities and (**e**,**f**) faradaic efficiencies for H_2_ and CO during chronoamperometric tests at −1.8 (left) and −2 V (vs. Ag/AgCl) (right) with L2, L20, M2 and M20 electrodes; (**a**,**b**) partial current densities normalized by the electrode geometric surface; (**c**,**d**) partial current densities normalized by the deposit surface areas reported in Table 1.

To analyze how the transition from a compact to a mossy morphology affects the catalytic activity towards the ERC, chronoamperometric tests were performed by using the L and M electrodes in an H-cell at two potentials (−1.8 V and −2.0 V vs. Ag/AgCl), with CO_2_ continuously bubbled into the catholyte (KHCO_3_, 0.5 M). The faradaic efficiencies (FEs) and partial current densities for CO and H_2_ are reported in Figure 8. To quantify the catalytic activity of the electrodes, the partial current densities were normalized by both the geometric electrode area (i.e., the projected two-dimensional surface area of the electrode) (Figure 8a,b) and the catalyst surface area (Figure 8e,f) measured via X-ray tomography (Table 1).

For all electrodes, the overall current density increased with the electrodeposition charge (Figure 8a–d), which can be attributed to the increased surface area of the deposits (Table 1). When comparing the partial current densities normalized by the geometric surface area (Figure 8a,b), no significant differences were found between the L and M electrodes at varying electrodeposition charges and electroreduction potentials, except at −2 V, where the M20 electrode exhibited a significantly larger H_2_ partial current density than that of L20. However, by analyzing the current data normalized by the catalyst surface area (Figure 8c,d), it is observed that the M electrodes show CO current densities larger than those of the L electrodes, indicating an improved intrinsic catalytic activity for the electroreduction of CO_2_ to CO. However, the M electrodes produced H_2_ in amounts slightly lower compared to those for CO, which contribute to reducing the CO FE.

Comparing the different morphologies, L2 and M2 show comparable CO FE values at the selected potentials. This result is consistent with the XRD characterization, which evidences the absence of relevant variations between the structures of the L2 and M2 electrodes (Figure 5). However, the indications from the XRD are contradicted for electrodes obtained with deposition charges equal to 20 C/cm^2^: a significant difference is found in the CO FEs between L20 and M20 at −1.8 V, despite the fact that no significant structural variation was revealed between the two electrodes by XRD. At −1.8 V, the CO FEs reached 60% for M20 and 82% for L20. The latter value was the highest CO FE recorded during the electrocatalytic tests (Figure 8). This result was compared to the literature values reported for nanoscale Zn catalysts in [36,86]. In accordance with these data sources, different zinc electrocatalyst formulations achieve CO FE values between 80% and 90% under optimized synthesis and electroreduction conditions, with only a few exceeding 90%. Accordingly, the 82% value achieved in the present study is in line with the highest values previously reported for nanoscale Zn catalysts.

The apparent contradiction between the XRD analysis and the electrocatalytic performances of L20 and M20 may be partly explained by the fact that, with the increasing transferred charge, the catalytic activity was initially governed by the structure of the metallic zinc phase, which was synthesized during the early electrodeposition stage and identified via XRD, but it was later influenced by the amorphous oxidized phase generated through zinc hydroxide precipitation. The latter phase does not significantly modify the structural information inferred from the XRD analysis, which is mainly reflected by the underlying crystalline metallic phase. This may explain the discrepancy between the XRD prediction and electrocatalytic performances and evidences the important role played by the oxidized zinc phase in determining the catalytic activity of electrodes synthesized at higher electrodeposition charges.

In contrast, the increased CO FE achieved with the L20 electrode compared to that of the M20 electrode at −1.8 V (Figure 8e), along with the observation that the M-type electrodes exhibited improved intrinsic activity (i.e., larger CO partial current density) for the ERC (Figure 8c,d), suggests that catalytic performances are influenced not only by the oxidized phase covering the zinc deposits. In fact, the same oxidized phase with an irregular morphology, which externally covered M2 and M20, was also present on the surface of L20. Therefore, the differences in the CO FEs between L20 and M20 should be attributed to factors other than the structure and composition of the external zinc phase.

Accordingly, an explanation for the improved selectivity to CO of the L20 electrode could be the formation of the zinc/oxidized zinc interface. To verify this hypothesis, the following results of the electrode characterization should be considered:

- No qualitative differences are observed between the XPS and Raman spectra of the different electrodes. The XPS confirms the presence of a Zn/ZnO heterointerface in all the samples, while the Raman spectroscopy reveals oxidized zinc species (zinc oxide and hydroxide).
- Qualitative differences are instead observed in the XRD spectra of L20 and M20: while M20 is completely amorphous, L20 exhibits crystalline characteristics, though they are less intense compared to L2. These results can be explained by the formation of an oxidized amorphous phase through zinc hydroxide precipitation. SEM image analysis confirms that the transition from metallic to oxidized zinc took place earlier in M20 than in L20.

In line with these results, the improved CO FE values cannot be attributed to qualitative differences in the chemical nature of the deposits: all electrodes contain metallic zinc and oxidized zinc phases yielding qualitatively identical XPS and Raman spectra. Instead, the variations in the electrocatalytic performances could be explained by differences in the relative proportions between the two zinc phases. Combining the results of the SEM and XRD analyses suggests that the zinc/zinc oxide interface plays a more important role in L20 than in M20. Several studies have demonstrated that these interfaces accumulate uncoordinated sites and oxygen vacancies, which can improve the selectivity to CO [66,67,87].

It is also worth noting that these characteristics may be further enhanced by the partial reduction of oxidized zinc species during the ERC. Indeed, the reduction, which proceeds through the dissolution and redeposition of zinc in metallic form, has been shown to increase the accumulation of defects and oxygen vacancies [36]. In contrast, we hypothesize that in the presence of highly irregular and filamentous oxidized zinc deposits, this reduction could negatively affect the catalytic activity. In this case, the reduction may start at the bottom of the filaments, causing the loss of zinc deposits. This could explain the reduced CO FE observed for M20 compared to that for L20.

The results of the present study provide a basis for the rational design of composite zinc/oxidized zinc electrodes for the ERC. Through the proposed electrochemical synthesis route, the catalytic activity can be varied by controlling the transition between zinc electrochemical reduction and zinc hydroxide precipitation. The optimization of this approach should aim at enhancing the zinc coverage on carbon paper and the control of the zinc-to-oxidized zinc ratio. For this purpose, the application of transient potentials initially enforcing the extensive and uniform nucleation of metallic zinc, followed by a shift towards the formation of the oxidized zinc phase, can be investigated.

## 4. Conclusions

Catalytic electrodes for CO_2_ electroreduction were fabricated by the galvanostatic electrodeposition of zinc onto carbon paper. The analysis shows that the transition from compact zinc nanostructures to composite zinc/oxidized zinc electrodes can be achieved by zinc electrochemical reduction followed by zinc hydroxide precipitation. This transition was achieved at selected current densities and zinc precursor concentrations by increasing the transferred charge. Notably, the transferred charge required to enforce the formation of zinc oxidized species can be increased by decreasing either the zinc precursor concentration or the current density, allowing the ratio between the metallic and oxidized zinc phases to be modified.

Different electrodes were selected to evaluate the impact of this ratio on the catalytic activity for the ERC. Interestingly, electrodes characterized by increased fractions of the oxidized zinc phase (M2, M20) yielded higher CO partial current densities per unit catalyst surface area, which suggests the presence of intrinsically more active sites. However, these electrodes exhibit lower CO FE values compared to those of electrodes with increased metallic zinc fractions (L2, L20). This evidences that M electrodes, mainly composed of amorphous zinc oxidized species in the form of flakes, provide a highly reactive surface for the reduction of CO_2_ to CO, likely due to the increased availability of defects, but they also effectively catalyze the HER.

A composite electrode (L20) with a well-developed zinc/zinc oxide interface (confirmed by XPS) yielded, in contrast, slightly lower CO production activity per unit catalyst surface area but significantly improved the selectivity. A CO FE equal to 82% was achieved with this electrode, which is in line with the highest values previously reported for zinc-based electrodes. It is proposed that this could be attributed to the accumulation of uncoordinated sites and oxygen vacancies induced by the Zn/ZnO heterointerface, which can effectively stabilize reaction intermediates and suppress the hydrogen evolution reaction.

These findings clearly evidence the importance of controlling the ratio between the metallic and oxidized zinc phases to optimize catalytic performances. In this regard, by elucidating the effect of the electrodeposition parameters on the transition from compact to mossy structures and evaluating the catalytic activity of the resulting morphologies, the illustrated analysis provides a basis for the rational synthesis of composite zinc/oxidized zinc electrodes for the ERC.

## Figures and Tables

**Table 1:** Surfaces and volumes of the M2, M20, L2 and L20 electrodes extracted from tomographic data. Reported data refer to the surfaces of the synthesized electrodes (4 cm^2^).

Electrode	Zinc Surface (mm^2^)	Zinc Volume (mm^3^)	Surface-to-Volume Ratio (mm^−1^)
M2	87	0.46	189
M20	120	1.14	105
L2	121	0.49	247
L20	202	1.18	171

**Table 2:** XPS Zn percent amount relative to total carbon of electrode samples.

Electrode	Zn (%)
**M2**	3.30
**M20**	3.75
**L2**	2.15
**L20**	3.75

## Data Availability

The raw data supporting the conclusions of this article will be made available by the authors upon request.

## Supplementary Materials

The following supporting information can be downloaded at https://www.mdpi.com/article/10.3390/nano15131025/s1, Figure S1: Frontal and isometric axonometry of electrodes L2 (a,a′); L20 (b,b′); M2 (c,c′); M20 (d,d′). Each of the reported views corresponds to a square section with a side length equal to 4.4 mm.
