# Supplementary material for: Analysis of Transition from Compact to Mossy Structures During Galvanostatic Zinc Electrodeposition and Its Implications for CO2 Electroreduction
Source: Nanomaterials (Basel). 2025 Jul 2;15(13):1025. doi: 10.3390/nano15131025 (PMC12251542; doi:10.3390/nano15131025)
Supplement: Supplementary file 1 [file nanomaterials-15-01025-s001.zip › Supporting Information.pdf]

Supplementary Materials

Analysis of the transition from compact to mossy structures during galvanostatic zinc electrodeposition and its implications for CO_2 electroreduction

Pietro Altimari[1a], Silvia Iacobelli[a], Pier Giorgio Schiavi[*a], Gianluca Zanellato[b], Francesco Amato[a], Andrea Giacomo Marrani[a], Olga Russina[a], Alessia Sanna[b], Francesca Pagnanelli[a]

[a]*Department of Chemistry, Sapienza University of Rome, Piazzale Aldo Moro 5, 00185, Rome, Italy*

[b]*Department of Basic and Applied Sciences for Engineering, Sapienza University of Rome, Piazzale Aldo Moro 5, 00185, Rome, Italy*

[1] Correspondence about this article should be addressed to Pietro Altimari (Pietro.altimari@uniroma1.it) and Pier Giorgio Schiavi (Piergiorgio.schiavi@uniroma1.it).

Tomographic reconstruction of the zinc electrodes

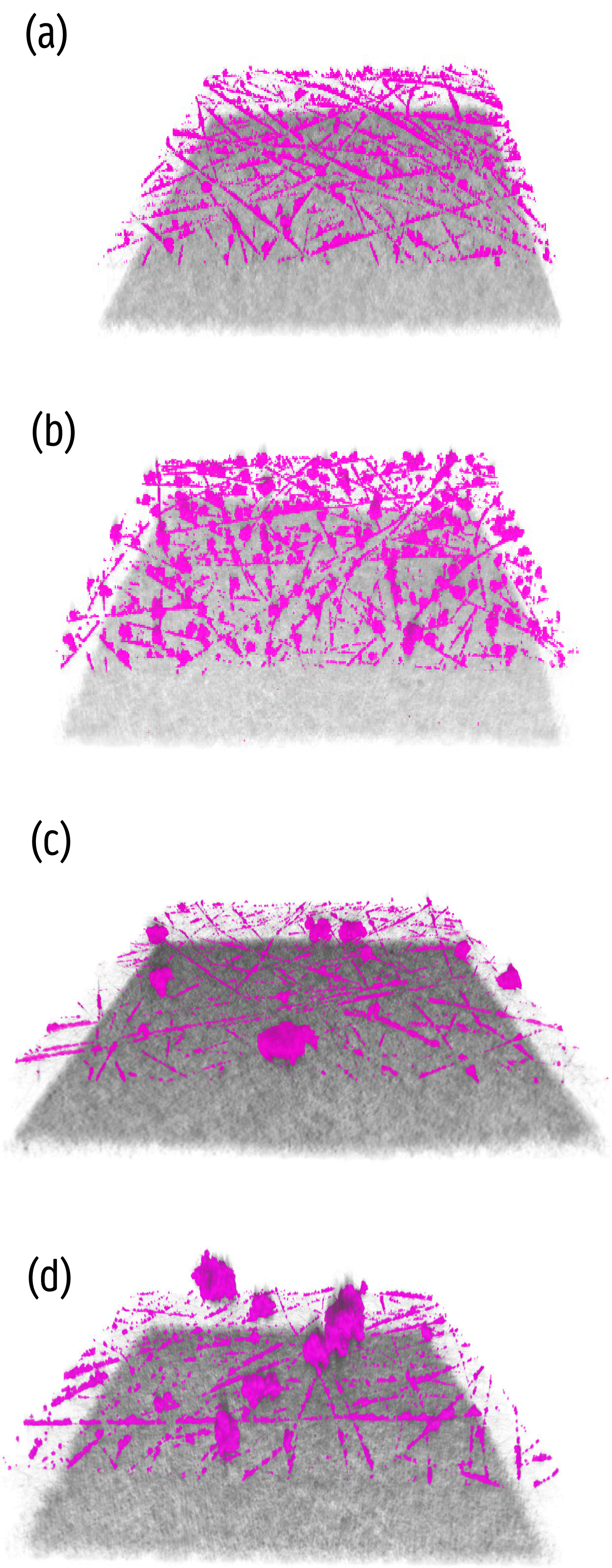

Figure S1 - Frontal and isometric axonometry of electrodes L2 (a), (a'); L20 (b), (b'); M2 (c), (c′); M20 (d), (d'). Each of the reported views corresponds to a square section with side length equal to 4.4 mm.
